# Social Participation and the Risk for Developing Mild Cognitive Impairment and Dementia Among Older Adults

**DOI:** 10.1093/geroni/igab046.049

**Published:** 2021-12-17

**Authors:** Hrafnhildur Eymundsdottir, Sigurveig Sigurdardottir, Alfons Ramel, Palmi Jonsson, Vilmundur Gudnason, Lenore Launer, Milan Chang

**Affiliations:** 1 The Icelandic Gerontological Research Institute, Gardabaer, Hofuoborgarsvaoio, Iceland; 2 University of Iceland, Reykjavik, Hofuoborgarsvaoio, Iceland; 3 University hospital Landspitali, Reykjavik, Hofuoborgarsvaoio, Iceland; 4 University of Iceland, Kopavogur, Hofuoborgarsvaoio, Iceland; 5 National Institute on Aging, Bethesda, Maryland, United States; 6 The Icelandic Gerontological Research Institute, Reykjavik, Hofuoborgarsvaoio, Iceland

## Abstract

Introduction: We aim to investigate the longitudinal associations between social participation and the risk of developing mild cognitive impairment (MCI|) and dementia over 5 years of follow-up among cognitively normal older adults. Methods: A total of 2802 participants had complete follow-up data from Age-Gene/Environment-Susceptibility-Reykjavik-Study. Social participation was assessed by a questionnaire asking the frequency of contact with children, relatives, friends and neighbors. MCI and dementia were diagnosed according to international guidelines and by a team composed of a geriatrician, neurologist, neuropsychologist, and neuroradiologist. Logistic regression analysis was used to assess the associations. Results: At baseline 8% (n=225) reported no social participation. Among cognitively normal participants at baseline, 5.6% (n=243) developed mild cognitive impairment and 2.4% (n= 103) developed dementia during a mean follow-up time of 5.2 years. After full adjustment with covariates including age, gender, education, marital status, vitamin D levels, depression and APOE ε4, those with no social participation at baseline were significantly more likely to develop MCI at follow-up (OR=1.953, P=0.001). However, social participation at baseline was not associated with higher dementia diagnosis at follow-up (OR= 1.490, P=0.194). Conclusions: Community-dwelling old adults who are socially inactive are more likely to develop MCI than those who are socially active. Social participation might independently indicate impending changes in cognitive function among older adults.

